# Mapping the evolution of artificial intelligence applications in bone metastasis: a bibliometric and visualization analysis

**DOI:** 10.3389/fonc.2026.1971840

**Published:** 2026-10-21

**Authors:** Yile Lin, Sihong Ye, Jie Wu, Yujia Hu, Haixiao Wu, Huining He, Jilong Yang, Yana Wang, Baicheng Ni, Liangkai Zhu, Chen Ding, Chun Liu, Elmar R. Musaev, Jun Wang, Chao Zhang, Ming Zheng

**Affiliations:** 1 Yueqing Hospital of Wenzhou Medical University, Wenzhou, China; 2 I. M. Sechenov First Moscow State Medical University (Sechenov University), Moscow, Russia; 3 Tianjin Medical University Cancer Institute and Hospital, National Clinical Research Center for Cancer, Key Laboratory of Cancer Prevention and Therapy, Tianjin’s Clinical Research Center for Cancer, Tianjin, China; 4 International Joint Laboratory of Ocular Diseases, School of Biomedical Engineering and Technology, Tianjin Medical University, Tianjin, China; 5 Federal State Autonomous Educational Institution of Higher Professional Education “Patrice Lumumba Peoples’ Friendship University of Russia” of the Ministry of Science And Higher Education of the Russian Federation, Moscow, Russia

**Keywords:** artificial intelligence, bibliometric analysis, bone metastasis, machine learning, precision oncology

## Abstract

**Background:**

Bone metastasis represents a major complication of advanced malignancies and is associated with substantial skeletal morbidity, impaired quality of life, and complex clinical decision-making. Artificial intelligence (AI) has rapidly emerged as an important computational approach for analyzing heterogeneous biomedical data. The developmental landscape, intellectual evolution, and future clinical directions of artificial intelligence applications in bone metastasis remain insufficiently characterized.

**Methods:**

A comprehensive bibliometric analysis was performed using publications retrieved from the Web of Science Core Collection, PubMed, and Scopus from January 2006 to June 2026. Bibliometric indicators, including annual publication trends, journal distribution, countries, institutions, authors, citation patterns, keyword co-occurrence, thematic evolution, and reference bursts, were analyzed using CiteSpace, VOSviewer, and R-based bibliometric tools.

**Results:**

A total of 540 publications involving 3,439 authors from 237 journals were identified. Research output increased substantially after 2020, with an annual growth rate of 24.01%, reflecting rapid expansion of AI-related investigations in bone metastasis. China contributed the largest number of publications, whereas the United States, Japan, and European countries showed substantial scientific influence and international collaboration. Thematic analysis demonstrated a progressive transition from early computer-aided diagnosis and bone scintigraphy analysis toward deep learning, radiomics, multimodal prediction, and language-based artificial intelligence applications. Imaging-related approaches remained the dominant research direction, while emerging studies increasingly focused on clinical information extraction, prognostic modeling, and integration of heterogeneous data sources. A small number of recent studies explored language-based AI for clinical information extraction, data organization, and knowledge support, suggesting an emerging but still limited extension beyond conventional imaging applications.

**Conclusion:**

AI research in bone metastasis has evolved from automated lesion recognition toward broader applications in disease characterization, risk assessment, and clinical decision support. Imaging, machine learning, deep learning, and radiomics remain the principal foundations of the field, whereas language-based AI currently represents a comparatively small and emerging research direction. Future studies should prioritize multicenter validation, multimodal data integration, prospective evaluation, and clinically meaningful endpoints. Large language model systems may warrant further investigation as potential components of future disease-specific AI frameworks, although their clinical utility in bone metastasis remains to be established.

## 1 Introduction

Bone metastasis represents a frequent and clinically consequential manifestation of advanced malignancies. It remains one of the most challenging complications encountered in oncology practice because of its irreversible impact on skeletal integrity, functional status, and patient quality of life. With continuous advances in systemic anticancer therapies, survival among patients with advanced malignancies has been substantially prolonged, resulting in a growing population living with metastatic disease. Bone is one of the most common sites of distant dissemination, particularly in breast cancer, prostate cancer, lung cancer, and renal cell carcinoma (1). Recent population-based studies demonstrated that synchronous bone metastasis occurs in a considerable proportion of patients with newly diagnosed solid tumors, with marked variations among different primary cancer types (2).Another population-based investigation further confirmed the heterogeneous distribution of bone metastasis across malignancies and highlighted the substantial clinical burden associated with skeletal dissemination (3). Although improvements in systemic treatment have enhanced disease control, bone metastasis continues to represent a major source of morbidity. Skeletal-related events (SREs), including pathological fractures, spinal cord compression, hypercalcemia, and cancer-associated bone pain, frequently lead to functional deterioration and reduced quality of life (4, 5).In clinical practice, the management of bone metastasis requires continuous evaluation of metastatic burden, skeletal complications, therapeutic response, and long-term prognosis. These complex clinical requirements have created an increasing demand for individualized approaches capable of integrating multidimensional clinical and biological information.

The assessment and management of bone metastasis increasingly rely on the interpretation of heterogeneous biomedical data. Medical imaging modalities, including computed tomography (CT), magnetic resonance imaging (MRI), positron emission tomography/computed tomography (PET/CT), and bone scintigraphy, remain fundamental tools for lesion detection, localization, and response evaluation (6). However, imaging findings represent only one component of the metastatic phenotype. Clinical characteristics, pathological parameters, treatment history, and survival outcomes provide complementary information for patient stratification and therapeutic decision-making. Advances in molecular profiling technologies have expanded the understanding of metastatic evolution by revealing genomic alterations, transcriptional programs, and tumor–microenvironment interactions involved in skeletal colonization (7, 8). The increasing complexity of these datasets has exposed limitations of conventional analytical approaches. Traditional statistical models often rely on predefined variables and may not fully capture nonlinear interactions among imaging characteristics, molecular changes, and clinical outcomes. Artificial intelligence (AI), including machine learning, deep learning, radiomics-based approaches, and large language models (LLMs) has therefore attracted increasing attention because of its ability to extract complex patterns from high-dimensional biomedical datasets (9–11). In oncology, AI-based methods have been increasingly explored as computational tools for precision medicine, supporting applications in diagnosis, risk prediction, prognosis estimation, and treatment optimization (12).

Within the field of bone metastasis, AI applications have expanded from image interpretation toward broader computational approaches for clinical and biological research. Imaging-based AI models have been widely investigated for automated detection, segmentation, and characterization of metastatic bone lesions. Deep learning approaches applied to CT, MRI, PET/CT, and bone scintigraphy have shown potential to improve diagnostic performance and reduce observer variability (13).Radiomics has further extended imaging analysis by converting medical images into quantitative features, enabling non-invasive assessment of tumor heterogeneity and metastatic characteristics (14). Beyond imaging, machine learning models have been developed for bone metastasis risk prediction and individualized prognostic assessment across different cancer populations (15). AI-based approaches have also been applied to survival prediction, treatment-response evaluation, and multi-omics analysis to explore metastatic progression and therapeutic resistance (16, 17).Collectively, these advances reflect a transition of AI in bone metastasis research from imaging-assisted diagnosis toward integrated computational frameworks for disease prediction and biological investigation.

Despite the rapid expansion of AI-related studies in bone metastasis, the overall research landscape remains incompletely characterized. Current investigations have been distributed across multiple cancer types, imaging platforms, computational algorithms, and clinical objectives. Research focusing on radiomics, deep learning-based imaging analysis, molecular prediction, and clinical risk modeling has largely evolved within relatively independent streams, making it difficult to determine the developmental trajectory and knowledge structure of this interdisciplinary field. In addition, several translational challenges remain unresolved, including limited external validation, dataset heterogeneity, algorithm interpretability, reproducibility, and uncertainty regarding clinical implementation (18, 19). Although many AI models have demonstrated promising performance in retrospective cohorts, their clinical applicability requires evaluation across diverse populations and real-world healthcare environments. A comprehensive understanding of scientific development patterns, influential contributors, emerging research directions, and collaborative structures is therefore essential for identifying current achievements and future opportunities in AI-assisted bone metastasis research.

Bibliometric analysis provides a quantitative framework for evaluating the development of scientific fields through systematic assessment of publication characteristics, citation patterns, collaboration networks, and knowledge structures (20). Compared with conventional narrative reviews, bibliometric approaches allow the visualization of research trajectories, identification of influential studies, recognition of emerging topics, and evaluation of collaboration patterns within rapidly evolving disciplines (21). Given the interdisciplinary characteristics of AI applications in bone metastasis, which integrate oncology, radiology, computer science, and molecular medicine, bibliometric analysis offers an appropriate approach for comprehensively evaluating the development and structure of this emerging research field.

In this study, a comprehensive bibliometric analysis was performed to characterize the global research landscape of artificial intelligence applications in bone metastasis. The analysis focused on publication trends, research productivity, international collaboration patterns, influential contributors, knowledge structures, and thematic evolution. By mapping the development trajectory of AI-assisted bone metastasis research, this study aimed to identify major research hotspots, clarify evolving scientific directions, and provide insights into future priorities for the application of artificial intelligence in metastatic bone disease.

## 2 Materials and methods

### 2.1 Data source

Relevant publications were systematically retrieved from three databases: the Web of Science Core Collection (WoSCC), PubMed, and Scopus. These databases were selected to provide broad coverage of multidisciplinary, biomedical, oncological, radiological, and artificial intelligence-related literature. WoSCC and Scopus provide extensive indexing of multidisciplinary scientific publications, whereas PubMed focuses primarily on biomedical and clinical research. The combined use of the three databases was therefore intended to improve literature coverage while reducing dependence on the indexing scope of any single database (22). All database searches were conducted on June 26, 2026, to minimize potential bias caused by daily database updates. The retrieval period extended from January 1, 2006, to June 26, 2026. Only English-language articles and reviews were considered eligible for inclusion. Because the search was completed on June 26, records published in 2026 represented a partial calendar year. These records were retained to capture the most recent developments but were interpreted cautiously in year-to-year publication trend analyses.

### 2.2 Search strategy

The search strategy was developed around two principal concept groups. The terms were formulated to capture both the disease domain and the major methodological categories used in AI-related bone metastasis research. The bone metastasis concept group was restricted to terms explicitly describing metastatic involvement of bone or the skeleton to maintain disease specificity. The AI concept group incorporated both broad AI terminology and commonly used methodological families, including machine learning, deep learning, radiomics, computer vision, computer-aided diagnosis, neural networks, support vector machines, random forests, and image segmentation, in order to capture studies that described specific computational methods without necessarily using the general term “artificial intelligence.” The first group represented bone metastatic disease and included the terms “bone metastasis,” “bone metastases,” “skeletal metastasis,” and “skeletal metastases.” The second group represented artificial intelligence and related computational methods. This group included terms associated with artificial intelligence, machine learning, deep learning, machine intelligence, computational intelligence, large language models, computer vision, computer-aided diagnosis, radiomics, artificial neural networks, convolutional neural networks, support vector machines, random forests, and image segmentation. Equivalent conceptual combinations were maintained across the three databases, while database-specific field tags and syntax were adapted according to the indexing structure of each platform. Database-specific search fields were used according to the indexing structure of each database. Topic searching was applied in WoSCC, Title/Abstract searching was used in PubMed, and TITLE-ABS-KEY searching was applied in Scopus. The search was restricted to articles and reviews published in English between 2006 and the search date. The complete search strategies for WoSCC, PubMed, and Scopus are provided in **Figure 1** and **Table 1**.

**Figure 1:**
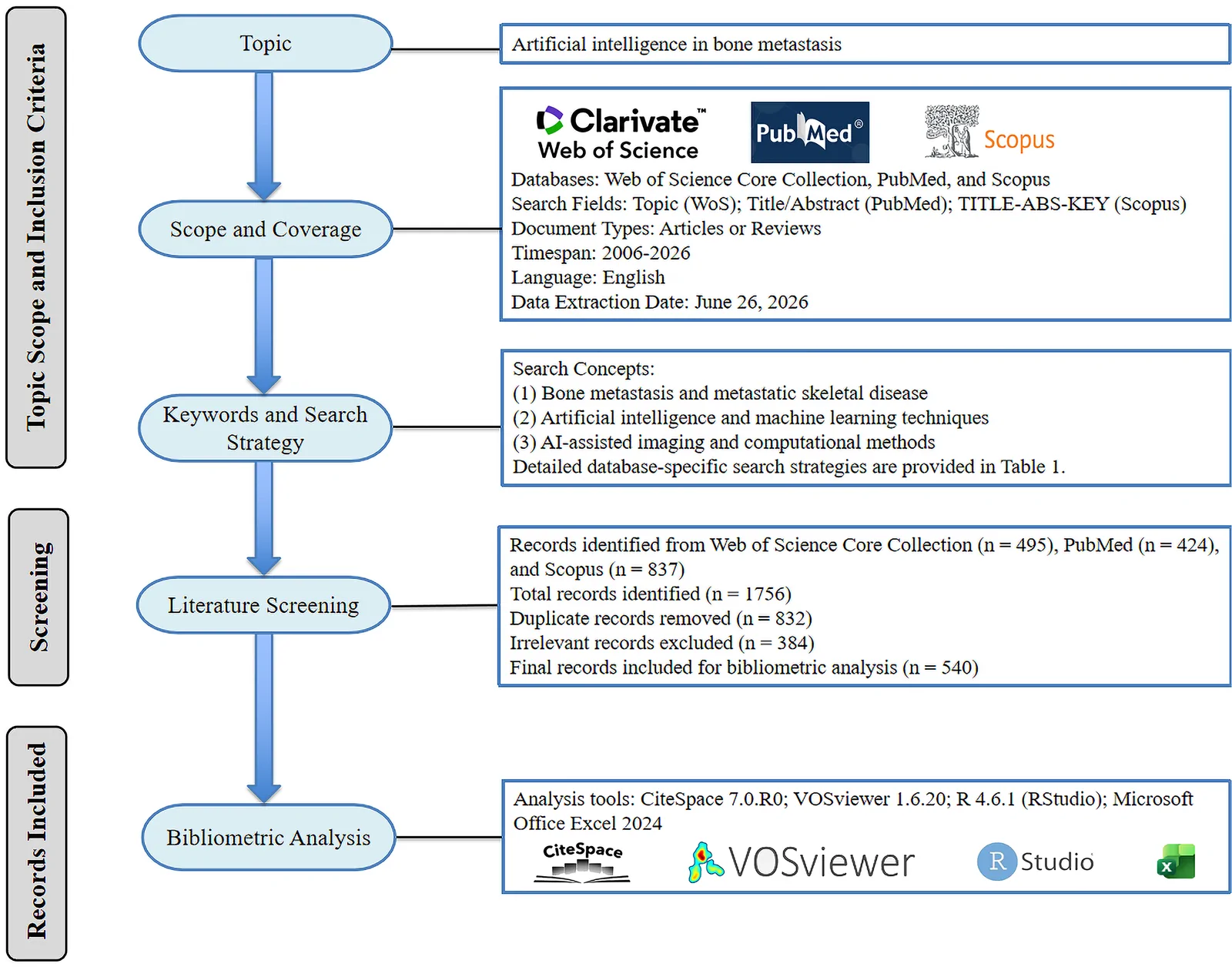
Workflow of literature retrieval, screening, and bibliometric analysis.

**Table 1:** Search strategies used in the bibliographic databases.

Database	Search strategy
Web of Science Core Collection	TS=((“bone metastasis” OR “bone metastases” OR “skeletal metastasis” OR “skeletal metastases”) AND (“artificial intelligence” OR “artificial-intelligence” OR “AI (Artificial Intelligence)” OR “machine learning” OR “deep learning” OR “deep learn*” OR “machine intelligence” OR “computational intelligence” OR “large language model*” OR “computer vision*” OR “computer-aided diagnosis” OR “radiomics” OR “artificial neural network” OR “convolutional neural network” OR “support vector machine” OR “random forest” OR “image segmentation”)) AND PY= (2006–2026) AND LA=(English) AND DT=(Article OR Review)
PubMed	((“bone metastasis”[Title/Abstract] OR “bone metastases”[Title/Abstract] OR “skeletal metastasis”[Title/Abstract] OR “skeletal metastases”[Title/Abstract]) AND (“artificial intelligence”[Title/Abstract] OR “artificial-intelligence”[Title/Abstract] OR “AI (Artificial Intelligence)”[Title/Abstract] OR “machine learning”[Title/Abstract] OR “deep learning”[Title/Abstract] OR “deep learn*”[Title/Abstract] OR “machine intelligence”[Title/Abstract] OR “computational intelligence”[Title/Abstract] OR “large language model*”[Title/Abstract] OR “computer vision*”[Title/Abstract] OR “computer-aided diagnosis”[Title/Abstract] OR “radiomics”[Title/Abstract] OR “artificial neural network”[Title/Abstract] OR “convolutional neural network”[Title/Abstract] OR “support vector machine”[Title/Abstract] OR “random forest”[Title/Abstract] OR “image segmentation”[Title/Abstract])) AND (“2006/01/01”[Date - Publication]: “2026/6/26”[Date - Publication]) AND English[Language] AND (Journal Article[Publication Type] OR Review[Publication Type])
Scopus	TITLE-ABS-KEY((“bone metastasis” OR “bone metastases” OR “skeletal metastasis” OR “skeletal metastases”) AND (“artificial intelligence” OR “artificial-intelligence” OR “AI (Artificial Intelligence)” OR “machine learning” OR “deep learning” OR “deep learn*” OR “machine intelligence” OR “computational intelligence” OR “large language model*” OR “computer vision*” OR “computer-aided diagnosis” OR “radiomics” OR “artificial neural network” OR “convolutional neural network” OR “support vector machine” OR “random forest” OR “image segmentation”)) AND PUBYEAR > 2005 AND PUBYEAR < 2027 AND LANGUAGE(English) AND (DOCTYPE(ar) OR DOCTYPE(re))

### 2.3 Literature screening and deduplication

The initial search identified 1,756 records, including 495 records from WoSCC, 424 records from PubMed, and 837 records from Scopus. Records retrieved from the three databases were exported and merged into a unified dataset using Microsoft Excel 2024. Because substantial overlap was expected across the three databases and bibliographic identifiers were not uniformly available for all records, a hierarchical deduplication procedure was applied. Duplicate publications were identified using available bibliographic identifiers, including the digital object identifier, PubMed identifier, accession number, article title, author information, publication year, and journal name. Exact matches based on DOI or other unique identifiers were removed first. For records without DOI information or with inconsistent identifiers, titles were compared after normalization. Records with identical or highly similar titles were further examined using author names, publication year, journal source, and other bibliographic information. Potential duplicates were removed only after concordance across these bibliographic fields was confirmed. A total of 832 duplicate records were removed, leaving 924 unique records for eligibility screening. Two reviewers independently screened the titles and abstracts according to the predefined inclusion and exclusion criteria. Studies were considered eligible if they met the following criteria: (1) the study directly addressed bone metastasis, metastatic bone lesions, skeletal metastasis, spinal metastasis, or another clearly defined form of metastatic bone involvement; (2) for original studies, at least one predefined AI-related method, such as machine learning, deep learning, computer vision, radiomics, computer-aided diagnosis, artificial neural networks, convolutional neural networks, support vector machines, random forests, or image segmentation, was directly applied to a bone metastasis-related task, including detection, identification, segmentation, classification, differential diagnosis, risk prediction, prognostic assessment, treatment-response evaluation, or quantitative analysis; for review articles, AI applications in bone metastasis were required to constitute a substantive focus of the review; and (3) the publication was an English-language article or review published between January 1, 2006, and June 26, 2026. Records were excluded if (1) bone metastasis was mentioned only as background information, previous medical history, a covariate, or an incidental finding without being an actual target of analysis; (2) AI-related methods were only mentioned or were applied to other diseases or outcomes rather than directly to a bone metastasis-related research task; (3) the study primarily investigated other bone diseases or lesions, such as primary bone tumors, osteoporosis, changes in bone mineral density, or local osseous invasion without metastatic bone disease as the study target; or (4) the record did not meet the predefined publication period, language, or document-type restrictions. When eligibility could not be determined from the title and abstract, the full text or additional bibliographic information was examined. Any disagreements or uncertain cases were reviewed by a third reviewer and resolved through discussion until consensus was reached. After screening, 384 irrelevant records were excluded. A final dataset of 540 publications was included in the bibliometric analysis. The literature retrieval and screening process is presented in **Figure 1** and **Supplementary Table S1**.

### 2.4 Data extraction and data standardization

Bibliographic information was exported from each database using the most complete available record format. The extracted variables included publication title, abstract, author names, author affiliations, country or region, journal title, publication year, document type, author keywords, indexed keywords, cited references, citation counts, DOI, and other available bibliographic identifiers. Because metadata structures differ among WoSCC, PubMed, and Scopus, the exported records were harmonized before analysis. Equivalent bibliographic fields were mapped into a common structure, and records with missing or inconsistent information were manually checked when necessary. For duplicated publications indexed in multiple databases, the most complete metadata were retained or supplemented using information from the corresponding records in the other databases.

Because citation coverage differed across databases, citation counts were harmonized using a predefined source hierarchy. WoSCC was used as the primary source for citation information. When the same publication was indexed in multiple databases, citation counts were not summed or averaged, and the WoSCC Times Cited value was retained. Records with missing citation data were manually verified using DOI, PMID, normalized title, author information, and publication year, and verified WoSCC citation counts were added when available. Records for which no WoSCC citation count could be confirmed were treated as missing rather than as zero citations. Scopus and PubMed were used to supplement other bibliographic metadata, but citation counts from different databases were not mixed in citation-based analyses.

Variants in author names, institutional names, country or region names, journal titles, and keywords were manually reviewed and unified when appropriate. For keyword normalization, singular and plural forms, spelling variants, abbreviations, hyphenated expressions, and closely related terms were merged. For example, terms such as “artificial intelligence” and “AI” were standardized where appropriate. Variants of “MRI,” “CT,” “PET/CT,” “bone metastasis,” and “bone metastases” were also reviewed to reduce artificial fragmentation in the keyword network. Generic terms with limited thematic value, database-generated indexing terms, and unrelated methodological expressions were removed when they did not contribute to interpretation of the research landscape. A thesaurus file for VOSviewer and an alias or term-merging file for CiteSpace were constructed to ensure consistency across network analyses. Institutional abbreviations and full names were merged only after manual verification. Country names were standardized using a unified naming convention. Author names were checked cautiously because identical surnames and initials may represent different researchers. Country, institution, and keyword variants were manually reviewed before merging, and non-institutional affiliation labels, such as departments or laboratories, were excluded where appropriate. Detailed standardization and term-merging rules were provided in the **Supplementary Tables S2**–**S4**.

### 2.5 Bibliometric analysis

Bibliometric analyses and visualizations were performed using R version 4.6.1 within the RStudio environment, VOSviewer version 1.6.20, and CiteSpace version 7.0.R0. Microsoft Office Excel 2024 was used for data tabulation and graphical formatting. R, primarily through the bibliometrix package, was used to analyze publication trends, source productivity and citation impact, contributions and collaboration patterns of countries, institutions, and authors, and keyword-related themes and trends. It was also used to generate three-field plots, Bradford’s Law distributions, SCP/MCP analyses, reference publication year spectroscopy, thematic maps, and temporal keyword analyses. Selected bibliometric data were exported to Microsoft Office Excel 2024 for graphical presentation and formatting.

VOSviewer was used to visualize country, institutional, and author collaboration networks, as well as keyword co-occurrence networks. Full counting was applied to all VOSviewer network analyses. For the country, institutional, and author co-authorship networks, the minimum number of documents was set to 5 and the minimum citation threshold was set to 0. For the institutional network, documents involving more than 25 organizations were excluded to reduce the influence of unusually large collaborative records. For keyword co-occurrence analysis, Author Keywords were used, and the minimum occurrence threshold was set to 5. Association-strength normalization was applied to the author and keyword networks. Where clustering was performed, the resolution parameter was set to 1.00, the minimum cluster size was set to 1, and merging of small clusters was enabled. Overlay visualizations of institutional and author collaboration networks were generated from the same underlying networks, with node color representing the average publication year. Node size represented publication output or keyword occurrence frequency, links indicated collaborative or co-occurrence relationships, and total link strength reflected the overall connectivity of each node.

CiteSpace was used to examine the intellectual structure and temporal evolution of artificial intelligence research in bone metastasis through journal dual-map overlay, reference citation-burst detection, keyword clustering, and timeline visualization. For the keyword clustering and timeline analysis, the time span was set to 2006–2026 with one year per slice. Author Keywords were used as the term source, and Keyword was selected as the node type. Node selection was based on the g-index with a scale factor of k=6. Pathfinder and pruning sliced networks were enabled to reduce redundant links and improve network interpretability.

## 3 Results

### 3.1 Annual publication trends and general characteristics

A total of 540 publications on artificial intelligence applications in bone metastasis were included from January 1, 2006, to June 26, 2026. Annual output remained low before 2020 but increased markedly thereafter, reaching a peak of 101 publications in 2025, the most recent complete calendar year. By June 26, 2026, 74 publications had been included during 2026. This value represents only part of the 2026 publication year (**Figure 2A**). The cumulative number of publications increased from 61 in 2019 to 540 by the retrieval date, indicating rapid expansion of the field in recent years (**Figure 2B**). The publications were distributed across 237 sources and involved 3,439 authors. The annual growth rate was 24.01%, and international co-authorship accounted for 19.63% of the dataset. The mean number of co-authors per publication was 8.13. A total of 1,118 author keywords and 16,020 references were identified. The mean document age was 3.46 years, with an average of 10.06 citations per publication (**Figure 2C**).

**Figure 2:**
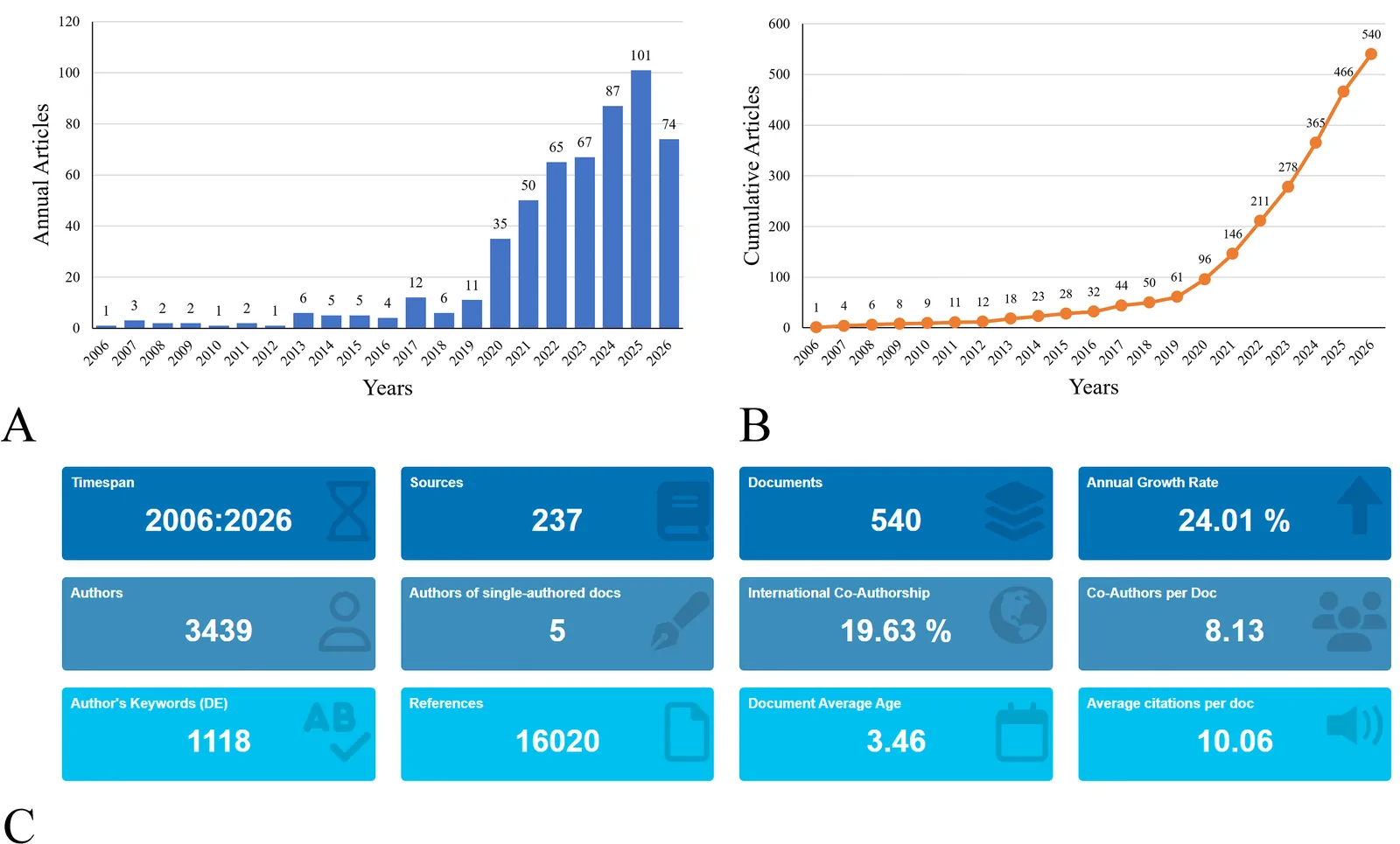
Temporal publication trends and descriptive characteristics of research on artificial intelligence in bone metastasis. **(A)** Annual publication output from 2006 to 2026. Data for 2026 include publications indexed through June 26, 2026, and therefore represent a partial calendar year. **(B)** Cumulative publication output during the study period. **(C)** Overview of the bibliometric dataset and its main descriptive indicators.

### 3.2 Analysis of journals

The 540 publications were distributed across 237 journals. Frontiers in Oncology was the most productive source, with 28 publications. It was followed by Annals of Nuclear Medicine with 21 publications. Cancers and Scientific Reports each contributed 17 articles, while Journal of Bone Oncology published 16. Among the leading sources, European Journal of Nuclear Medicine and Molecular Imaging received the highest citation count, with 365 citations from seven publications. This pattern suggests that publication volume and citation influence were not fully aligned across journals (**Figure 3A**). Annals of Nuclear Medicine ranked first by H-index, with a value of 11. Cancers and Frontiers in Oncology followed, both with an H-index of 9. An H-index of 7 was observed for Diagnostics, Journal of Bone Oncology, and Scientific Reports (**Figure 3B**). The prominence of nuclear medicine, oncology, and imaging journals reflects the current clinical orientation of this field. Much of the literature remains centered on image-based detection, lesion characterization, and predictive modeling. According to Bradford’s law, 14 journals were classified as core sources. Zone 2 included 57 journals, whereas Zone 3 contained 166 journals. The literature was therefore widely dispersed, although a relatively small group of journals formed the main publication core (**Figure 3C**). The dual-map overlay showed that the citing literature was concentrated in medicine and clinical research. The cited knowledge base was located mainly in health, nursing, medicine, molecular biology, and genetics (**Figure 3D**).

**Figure 3:**
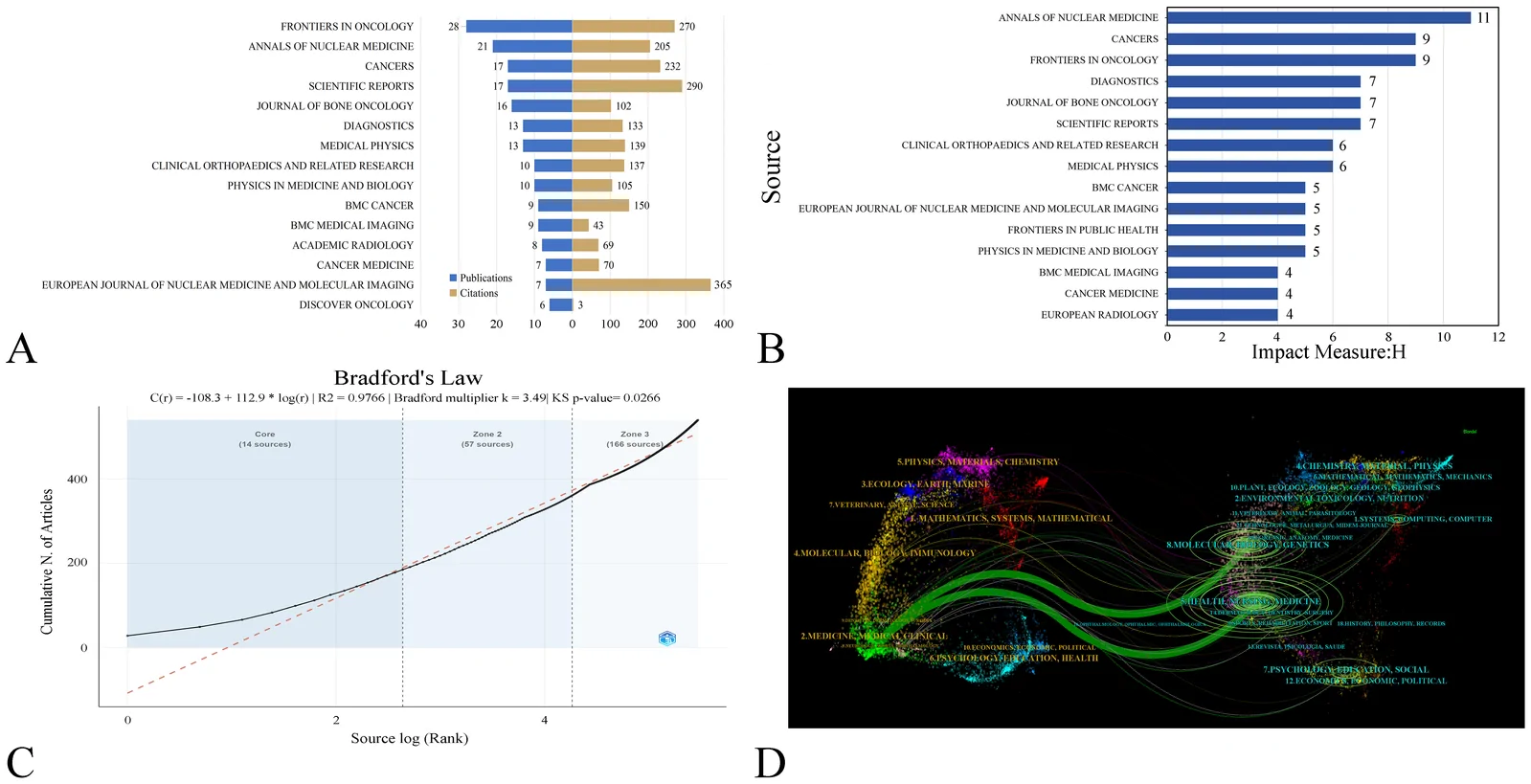
Journal productivity, impact, and citation structure in research on artificial intelligence in bone metastasis. **(A)** Publication output and citation counts of the leading journals. **(B)** Journals with the highest h-index values. **(C)** Core and non-core journals identified according to Bradford’s Law. **(D)** Dual-map overlay of journal disciplines, with citing journals shown on the left and cited journals shown on the right.

### 3.3 Analysis of countries

National scientific output increased markedly after 2020, with China showing the most pronounced rise. The United States and Japan also maintained substantial contributions, whereas growth from European countries was more moderate (**Figure 4A**). China ranked first with 233 publications, followed by Japan and the United States, each with 51 publications. Italy and South Korea contributed 19 and 18 publications, respectively (**Table 2**). The international collaboration network was centered mainly on China, the United States, Japan, Italy, and South Korea. Dense links were also observed between these countries and several European research groups (**Figure 4B**). The three-field plot further illustrated the relationships among institutions, authors, and countries. Most leading authors were affiliated with Chinese institutions, while additional contributions originated from the United States, Australia, Japan, and several European countries (**Figure 4C**). Single-country publications remained dominant in most leading countries. China produced 204 single-country and 29 multiple-country publications. Japan contributed 44 single-country and 7 multiple-country publications. The United States showed a more balanced collaboration profile, with 31 single-country and 20 multiple-country publications. The Netherlands also had a relatively high proportion of international publications, with six multiple-country publications among 13 articles (**Figure 4D** & **Table 2**). The geographical distribution confirmed that research activity was concentrated in East Asia, North America, and Western Europe. Contributions from Africa, South America, and several parts of Central Asia remained limited (**Figure 4E**).

**Figure 4:**
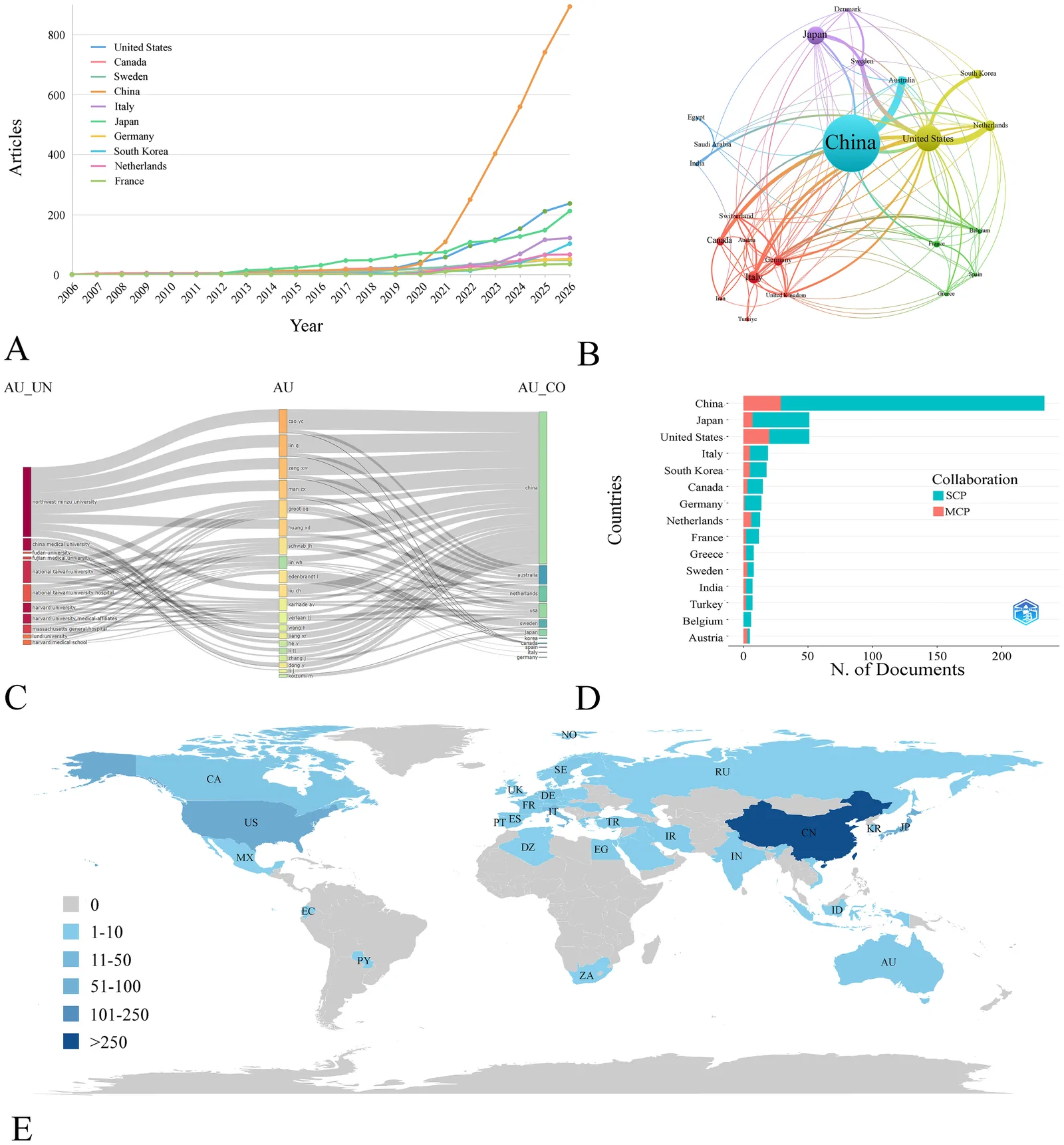
Country-level research output and international collaboration in research on artificial intelligence in bone metastasis. **(A)** Publication-output trends of the 10 most productive countries from January 1, 2006, to June 26, 2026. **(B)** Country co-authorship network. **(C)** Three-field plot linking affiliations, authors, and countries. **(D)** Proportions of single-country publications (SCPs) and multiple-country publications (MCPs) by country. **(E)** Global distribution of publications according to country-level research output.

**Table 2:** Country-level research performance and collaboration patterns.

Rank	Country	Articles	Frequency	TC	AC/P	SCP	MCP
1	China	233	893	1933	8.3	204	29
2	Japan	51	212	730	14.3	44	7
3	United States	51	237	766	15.0	31	20
4	Italy	19	122	152	8.0	14	5
5	South Korea	18	103	163	9.1	13	5
6	Canada	15	52	177	11.8	12	3
7	Germany	14	51	133	9.5	13	1
8	Netherlands	13	67	52	4.0	7	6
9	France	12	35	151	12.6	10	2
10	Greece	8	26	242	30.2	6	2

AC/P, average citations per publication; TC, total citations; SCP, single-country publications; MCP, multiple-country publications. Frequency: frequency of country occurrence in the dataset.

### 3.4 Analysis of institutions

Northwest Minzu University was the most productive institution, with 64 publications, followed by China Medical University with 47. Harvard University and Harvard University Medical Affiliates contributed 34 and 31 publications, respectively. China Medical University (Taiwan, China), National Taiwan University, and the University of Toronto each produced 28 publications (**Figure 5A**). Institutional output increased mainly after 2020. Northwest Minzu University showed the most pronounced recent growth and reached 64 publications by 2026. China Medical University also expanded rapidly, particularly after 2022. More gradual increases were observed for Harvard University, Harvard University Medical Affiliates, Massachusetts General Hospital, and National Taiwan University (**Figure 5B**). The collaboration network revealed several institution-centered clusters rather than a single integrated global network. China Medical University was one of the major collaborative hubs and was connected with multiple Chinese and international institutions, including Fudan University, Fujian Medical University, Nanchang University, Tianjin Medical University, Sichuan University, Sun Yat-sen University, and Johns Hopkins University. Northwest Minzu University formed another prominent cluster, showing close collaboration with Charles Sturt University and Lanzhou University. An independent international cluster was centered on Harvard Medical School and Massachusetts General Hospital, with additional links to University Medical Center Utrecht. Smaller collaborative groups were also observed among Lund University, the University of Gothenburg, and Sahlgrenska University Hospital, as well as among several institutions in Taiwan (**Figure 5C**). The overlay visualization showed that several high-output institutions became active relatively recently. Northwest Minzu University, Charles Sturt University, and parts of the China Medical University network were associated with later average publication years. Harvard Medical School and the University of Toronto represented comparatively earlier contributors (**Figure 5D**).

**Figure 5:**
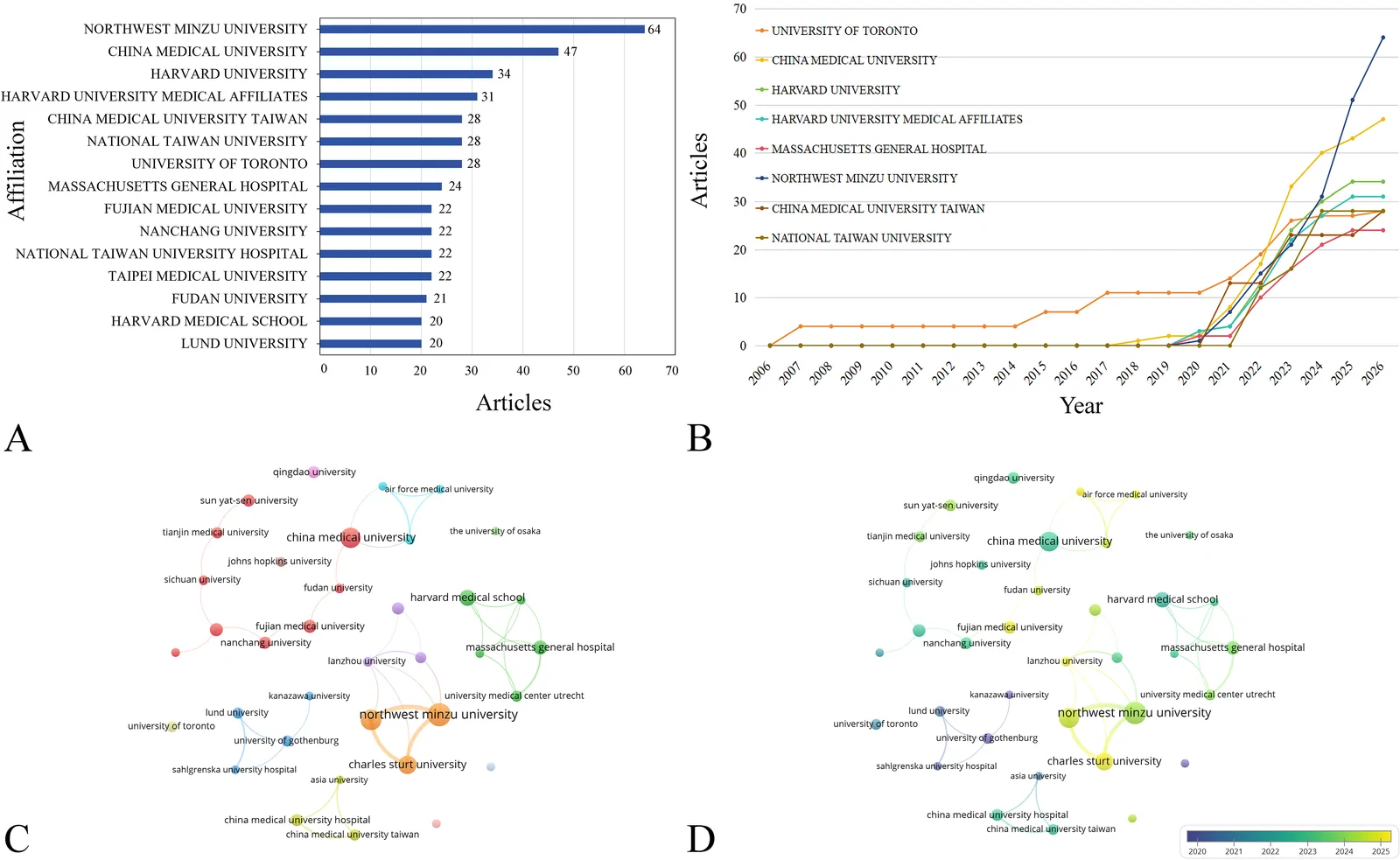
Institutional productivity and collaboration in research on artificial intelligence in bone metastasis. **(A)** The 15 most productive institutions ranked by publication output. **(B)** Publication-output trends of the leading institutions from January 1, 2006, to June 26, 2026. **(C)** Institutional collaboration network. **(D)** Temporal overlay of the institutional collaboration network.

### 3.5 Analysis of authors

The publication trajectories of the leading authors are shown in **Figure 6A**. Most high-output researchers became active after 2020. Cao YC was the most productive author, with 22 publications, followed by Lin Q with 21 and Zeng XW with 20. Man ZX and Huang XD contributed 18 and 15 publications, respectively. Cao YC and Lin Q also recorded the highest local citation counts among the most productive authors, with 84 citations each (**Table 3**). The co-authorship network showed several distinct research groups rather than a single integrated community (**Figure 6B**). A dense cluster was formed by Cao YC, Lin Q, Zeng XW, Man ZX, and Huang XD. Another group included Groot OQ, Schwab JH, and related collaborators. Smaller and relatively isolated clusters were also observed, which suggests that collaboration remained concentrated within established teams. The overlay visualization indicated that the largest author cluster was mainly active during the more recent phase of the field, particularly from 2023 onward (**Figure 6C**). Edenbrandt L showed the highest citation impact, with 353 total citations and 96 local citations, despite publishing only 10 articles. Cao YC and Edenbrandt L shared the highest H-index of 8. Schwab JH also showed substantial academic impact, with 193 citations and an H-index of 7 (**Table 3**).

**Figure 6:**
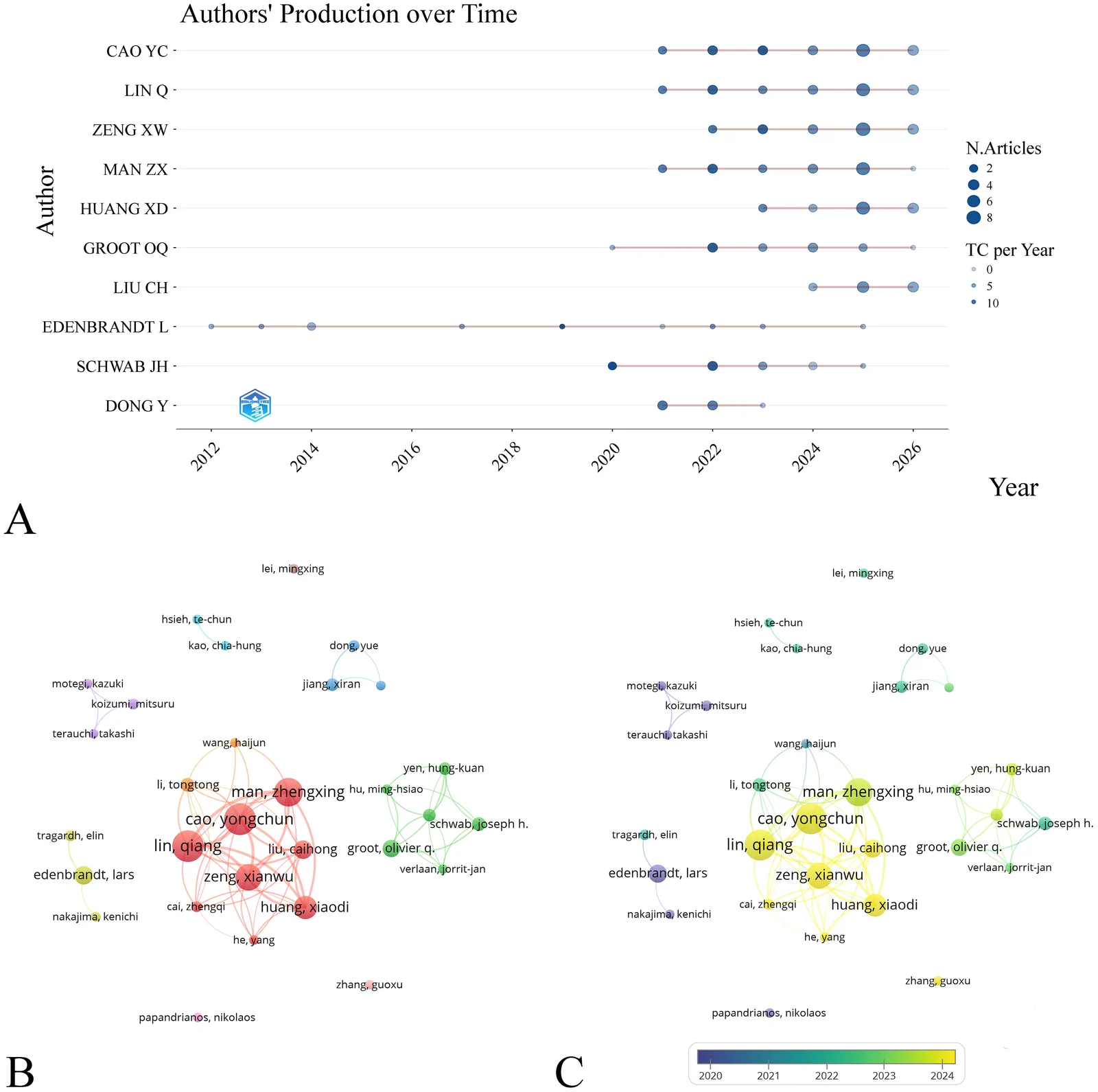
Author productivity and collaboration in research on artificial intelligence in bone metastasis. **(A)** Publication-output trends of the 10 most productive authors. **(B)** Author co-authorship network. **(C)** Temporal overlay of the author co-authorship network.

**Table 3:** Author-level scientific performance and impact.

Rank	Author	Documents	Local citations	Citations	H-index
1	CAO YC	22	84	201	8
2	LIN Q	21	84	182	7
3	ZENG XW	20	54	135	6
4	MAN ZX	18	83	178	7
5	HUANG XD	15	25	62	5
6	GROOT OQ	12	22	130	7
7	LIU CH	11	10	27	4
8	EDENBRANDT L	10	96	353	8
9	SCHWAB JH	10	36	193	7
10	DONG Y	7	28	105	6

### 3.6 Influential references and citation bursts

Citation-burst analysis identified a series of publications that attracted intensive attention over relatively short periods (**Figure 7A**). The strongest burst was observed for Horikoshi et al.’s study on computer-aided diagnosis of bone scintigrams, with a burst strength of 7.69 from 2013 to 2017. Early influential references were largely related to the Bone Scan Index and automated quantification of skeletal tumor burden. More recent bursts shifted toward deep learning and image-based classification. The review by Litjens et al. showed a burst strength of 6.42 during 2020-2021. Two studies on deep-learning-based bone metastasis classification remained active from 2021 to 2023. The most locally cited publication was “Global Cancer Statistics 2020: GLOBOCAN Estimates of Incidence and Mortality Worldwide for 36 Cancers in 185 Countries”, with 46 local citations. The U-Net related paper and the study on computer-assisted interpretation of planar whole-body bone scans each received 41 local citations. Other highly cited references addressed metastatic bone disease, Bone Scan Index quantification, radiomics, and deep learning in medical image analysis (**Figure 7B**; **Table 4**). The annual number of cited references remained low during the early period and increased sharply after 2010. A clear peak was reached around 2021. The deviation from the five-year median was highest in 2020, which marked a notable increase in citation activity (**Figure 7C**).

**Figure 7:**
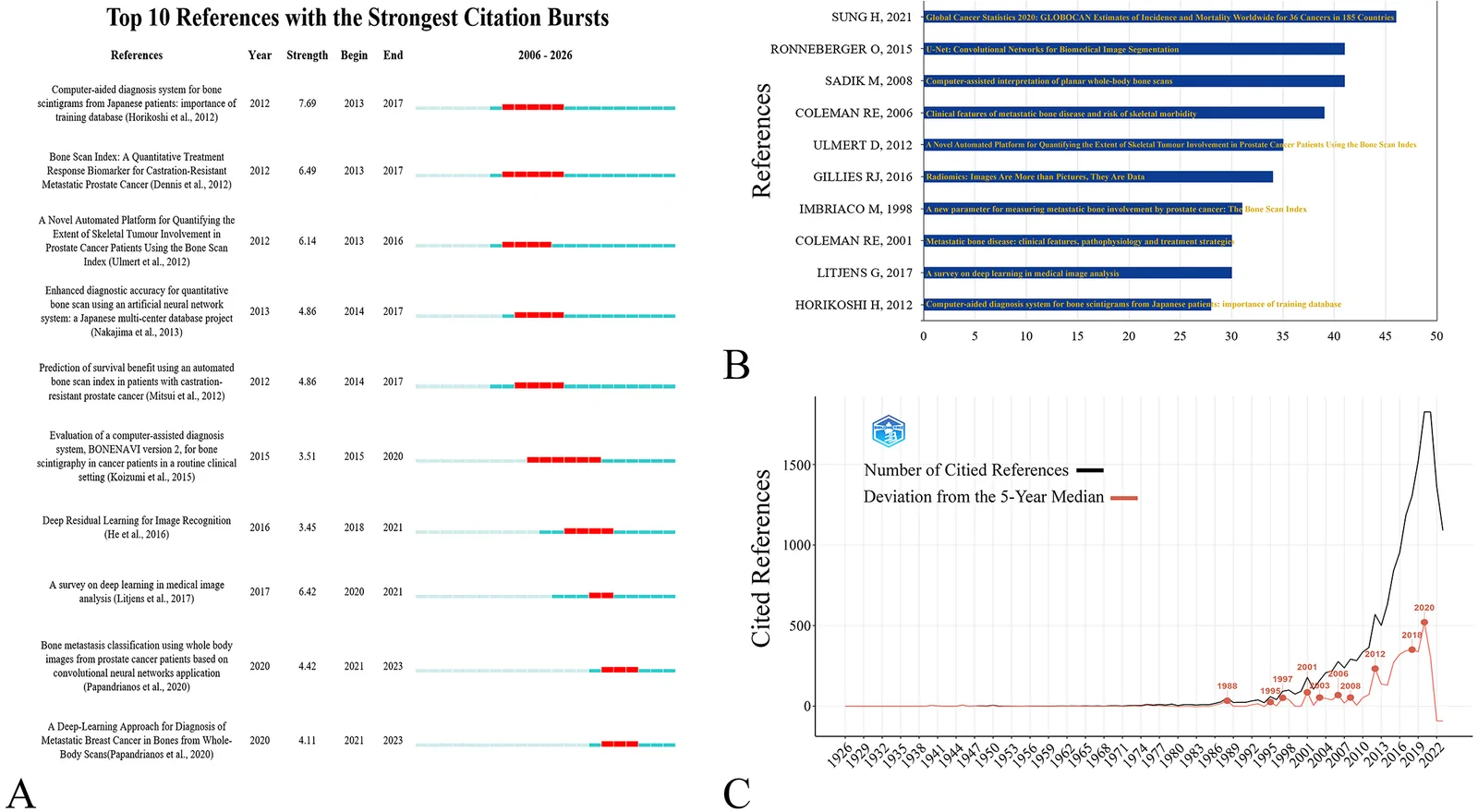
Citation structure and intellectual foundations of research on artificial intelligence in bone metastasis. **(A)** The 10 references with the strongest citation bursts. **(B)** The 10 references with the highest local citation counts. **(C)** Reference publication year spectroscopy.

**Table 4:** Top 10 influential publications ranked by local citations.

Rank	Title	Source	Year	First author	IF(2026)/JCR	Local citations
1	Global Cancer Statistics 2020: GLOBOCAN Estimates of Incidence and Mortality Worldwide for 36 Cancers in 185 Countries	CA: A Cancer Journal for Clinicians	2021	Hyuna Sung	685.2/Q1	46
2	U-Net: Convolutional Networks for Biomedical Image Segmentation	Lecture Notes in Computer Science	2015	Olaf Ronneberger	N/A	41
3	Computer-assisted interpretation of planar whole-body bone scans	Journal of Nuclear Medicine	2008	May Sadik	9.6/Q1	41
4	Clinical features of metastatic bone disease and risk of skeletal morbidity	Clinical Cancer Research	2006	Robert E Coleman	10.9/Q1	39
5	A Novel Automated Platform for Quantifying the Extent of Skeletal Tumor Involvement in Prostate Cancer Patients Using the Bone Scan Index	European Urology	2012	David Ulmert	29.1/Q1	35
6	Radiomics: Images Are More than Pictures, They Are Data	Radiology	2016	Robert J Gillies	17.6/Q1	34
7	A new parameter for measuring metastatic bone involvement by prostate cancer: The Bone Scan Index	Clinical Cancer Research	1998	M Imbriaco	10.9/Q1	31
8	Metastatic bone disease: clinical features, pathophysiology and treatment strategies	Cancer Treatment Reviews	2001	R.E. Coleman	10.6/Q1	30
9	A survey on deep learning in medical image analysis	Medical Image Analysis	2017	Geert Litjens	14.0/Q1	30
10	Computer-aided diagnosis system for bone scintigrams from Japanese patients: importance of training database	Annals of Nuclear Medicine	2012	Hiroyuki Horikoshi	2.4/Q2	28

### 3.7 Keyword clustering and thematic evolution

Keyword co-occurrence analysis identified four major thematic clusters (**Figure 8A**). Cluster 1 was centered on bone metastasis, machine learning, radiomics, prognostic modeling, and treatment-related assessment. Cluster 2 was dominated by deep learning, bone scintigraphy, image classification, and computer-aided diagnosis. Cluster 3 mainly involved prostate cancer and multimodal imaging, including magnetic resonance imaging and positron emission tomography. Cluster 4 reflected broader methodological development, with terms such as artificial intelligence, convolutional neural networks, image segmentation, and natural language processing. The timeline visualization further divided the literature into seven thematic groups (**Figure 8B**). Early work was largely based on computer-aided diagnosis, bone scintigraphy, and quantitative skeletal assessment. More recent clusters focused on deep-learning-based image analysis, bone metastasis imaging, predictive modeling, and cancer-specific applications. Prostate cancer formed a sustained research branch, whereas breast cancer-related bone metastasis remained smaller and comparatively less developed. The thematic map showed that deep learning, bone scintigraphy, and convolutional neural networks had relatively high density and centrality, indicating well-developed and influential themes (**Figure 8C**). Artificial intelligence, immunotherapy, and biomarkers appeared as more specialized themes. Radiomics, breast cancer, and computed tomography were positioned as basic themes with broad relevance but comparatively limited internal development. Several imaging-related topics, including prostate cancer, positron emission tomography, and whole-body magnetic resonance imaging, occupied the emerging or declining quadrant. Their position should be interpreted cautiously because it may reflect either reduced recent attention or a field that has not yet reached stable development. The keyword heatmap confirmed a marked shift in research focus over time (**Figure 8D**). Bone metastasis remained the dominant term, with 216 occurrences. Machine learning and deep learning followed with 106 and 103 occurrences, respectively. Radiomics, prostate cancer, artificial intelligence, and bone scintigraphy were also frequently represented (**Table 5**). Notably, language-based AI did not constitute a major high-frequency theme in the current dataset. Although natural language processing appeared within the broader methodological cluster, LLM-related applications remained comparatively sparse relative to machine learning, deep learning, radiomics, and imaging-based approaches.

**Figure 8:**
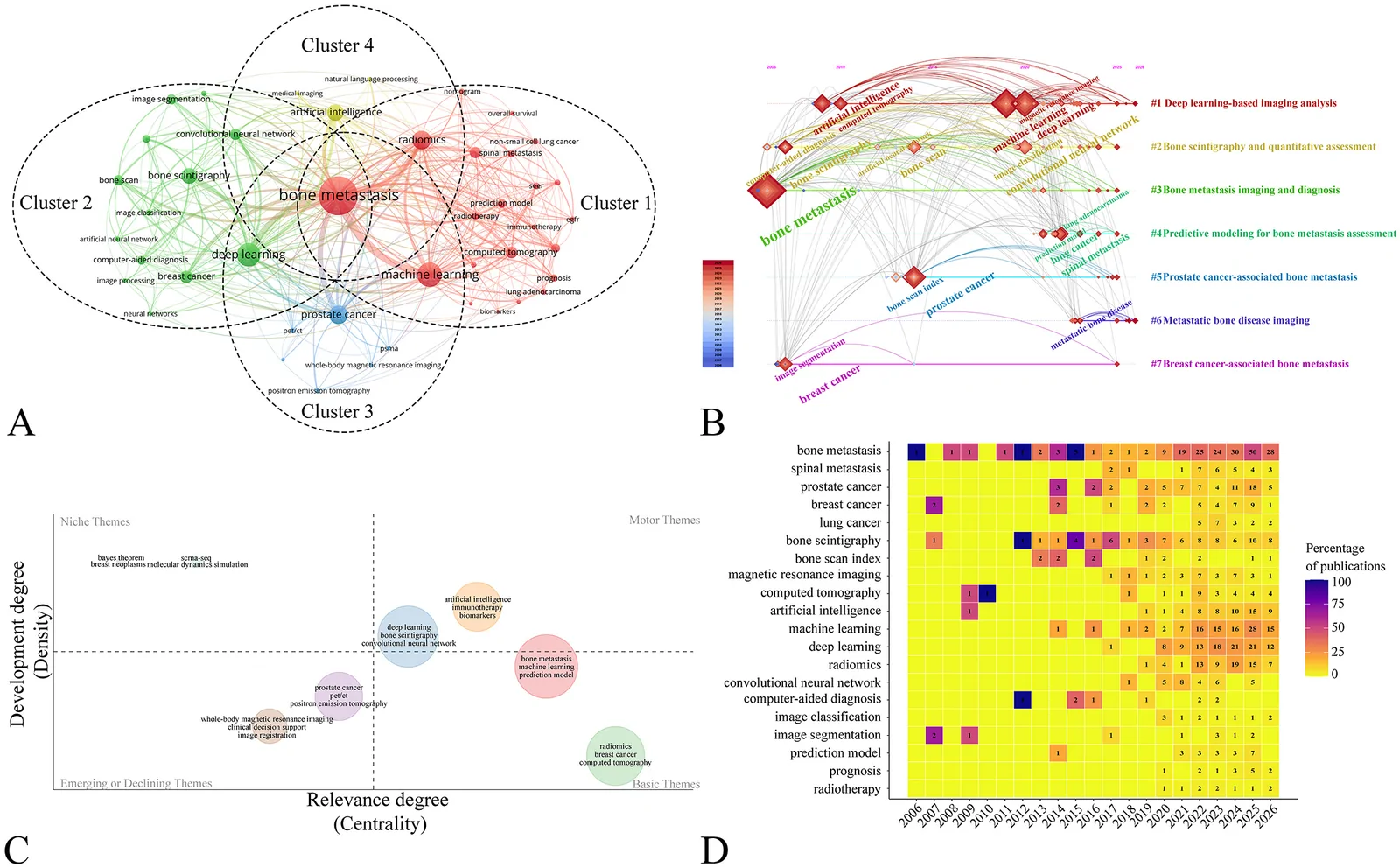
Keyword evolution and thematic structure of research on artificial intelligence applied to bone metastasis. **(A)** Keyword co-occurrence network showing four major thematic clusters. **(B)** Timeline visualization of seven keyword clusters. **(C)** Thematic map based on cluster centrality and density. **(D)** Annual heatmap of keyword use from January 1, 2006, to June 26, 2026.

**Table 5:** Top 15 most frequent author keywords.

Rank	Terms	Frequency
1	bone metastasis	216
2	machine learning	106
3	deep learning	103
4	radiomics	69
5	prostate cancer	68
6	artificial intelligence	57
7	bone scintigraphy	51
8	breast cancer	35
9	computed tomography	31
10	spinal metastasis	31
11	convolutional neural network	30
12	magnetic resonance imaging	29
13	bone scan	26
14	image segmentation	24
15	prediction model	20

## 4 Discussion

This study provides a comprehensive overview of artificial intelligence applications in bone metastasis research from 2006 to June 2026. A total of 540 publications were identified across WoSCC, PubMed, and Scopus. Research activity increased markedly after 2020, while imaging-based applications remained the dominant component of the field. The intellectual structure gradually expanded from computer-assisted interpretation and quantitative assessment of skeletal tumor burden toward deep learning, radiomics, prognostic modeling, and emerging language-based applications. China contributed the largest publication volume, whereas the United States, Japan, and several European countries maintained substantial citation-based visibility within the bibliometric network. Overall, the field is expanding rapidly, but international collaboration and clinical translation remain limited.

### 4.1 Publication growth, geographic distribution, and academic influence

The sharp rise in publications after 2020 probably reflects the convergence of several methodological and infrastructural developments. Krizhevsky et al. established the practical value of graphics-processor-trained convolutional neural networks (CNNs) for large-scale image classification in 2012 (23). Ronneberger et al. subsequently introduced U-Net for biomedical image segmentation (24). Vaswani et al. later described the transformer architecture (25). During the same period, deep-learning tools became more accessible and radiomics workflows became increasingly standardized (26). These developments enabled quantitative information to be extracted from CT, MRI, PET/CT, and bone scintigraphy at a scale beyond conventional visual assessment. Representative studies extended automated analysis to whole-skeleton segmentation, three-dimensional spinal-lesion characterization, and multiresolution MRI detection (27–29). The rise in cited references around 2020 and the citation peak in 2021 support the interpretation that this was a substantive methodological transition. Computer-aided analysis was being replaced by more data-intensive representation learning. Pi et al. exemplified this change by training an attention-augmented multiview network on 15,474 anterior and posterior bone-scan examinations from 13,811 patients (30). The 2026 publication count was incomplete at the time of retrieval because the search was conducted on June 26, 2026. The apparent change from 2025 to 2026 should not be interpreted as a temporal decline. The 2026 records were retained to capture the most recent research topics, but they were not treated as directly comparable with complete calendar years when interpreting annual publication trends. The most recent publications also suggest that AI applications are beginning to extend beyond conventional imaging tasks toward clinical text processing, information extraction, and computational modeling. These emerging applications remain limited in number, but they indicate a broadening of the data types and clinical questions addressed by AI in bone metastasis research (31–33).

China ranked first in publication volume and showed the most pronounced recent growth. This pattern may reflect the country’s large patient population, growing access to hospital-based multimodal data, and rapid expansion of AI research groups (34). Publication volume, however, did not consistently correspond to citation influence. The United States showed a more balanced profile of domestic and international work. Several North American institutions also contributed earlier and more highly cited studies. China’s recent advances in AI infrastructure may help sustain its publication advantage and support more influential clinical research. This outcome is not assured by scale alone. Zhang et al. developed a clinically applicable system for detecting and diagnosing bone metastases on CT. Development and validation were conducted across five hospitals, followed by evaluation in a large real-world cohort (35). This study illustrates how publication volume can be converted into stronger evidence through multicenter testing and deployment-oriented evaluation. China and the United States are often presented as competitors in AI. In practice, shared methods and cross-system validation may be more important for solving clinically relevant problems (29). National performance should therefore be judged by methodological contribution, external validation, citation influence, and international collaboration, not publication counts alone.

International co-authorship accounted for only 19.63% of the included literature, indicating that cross-border collaboration remains limited. Existing networks were concentrated around China, the United States, Japan, Italy, South Korea, and several European countries. Contributions from Africa, South America, and parts of Central Asia were comparatively scarce. This imbalance may constrain dataset representativeness and delay clinical translation across different health-care systems. Medical AI requires resources that extend well beyond algorithm design. High-performance computing, expert annotation, data harmonization, cybersecurity, software maintenance, and prospective validation all require sustained investment (36). These requirements are difficult to meet within isolated institutions. International and multicenter collaboration can distribute costs and expose models to variation in scanners, populations, disease phenotypes, and clinical workflows (37, 38). Wang et al. combined radiological and computer-science expertise across China and the United States (29). Their study became the most highly cited contribution in the present corpus. The Japanese multicenter study reported by Nakajima et al. likewise showed how pooled institutional data can support calibration and external validation (39). International collaboration may facilitate access to heterogeneous populations, imaging platforms, and clinical environments, thereby creating opportunities for broader validation. However, collaboration metrics themselves describe network structure and should not be interpreted as direct evidence of methodological quality, model reliability, or clinical significance.

### 4.2 Journals, influential contributors, and the intellectual foundation

Bibliometric indicators should be interpreted as measures of scholarly activity and visibility rather than direct measures of research quality or clinical importance. Publication counts primarily reflect productivity, whereas citation counts and H-index values capture citation-based influence within a given time window. These metrics are affected by publication age, citation latency, field size, topic popularity, journal visibility, and citation practices. Accordingly, highly cited studies are not necessarily methodologically superior or more clinically relevant, while recently published studies may remain underrepresented because insufficient time has elapsed for citations to accumulate. Collaboration metrics similarly describe the structure and extent of research networks rather than the quality of the resulting evidence. The distribution of publications across 237 journals highlights the multidisciplinary nature of AI research in bone metastasis. Leading sources span oncology, nuclear medicine, radiology, imaging science, and general medical research. Frontiers in Oncology published the largest number of articles, whereas European Journal of Nuclear Medicine and Molecular Imaging showed greater citation influence despite a smaller publication volume. This citation-based prominence reflected bibliometric visibility within the dataset and should not be interpreted as a direct measure of journal quality or the clinical importance of individual studies. Annals of Nuclear Medicine had the highest H-index among the leading journals. The H-index similarly combines publication productivity and citation accumulation but does not directly evaluate methodological rigor, external validity, reproducibility, or clinical impact. The prominence of nuclear medicine journals reflects the historical importance of bone scintigraphy in automated skeletal assessment. Its whole-body coverage and relatively standardized planar format provided a practical foundation for early computer-aided analysis and quantitative measurement of metastatic burden (40, 41). The subsequent expansion of the literature across oncology and imaging journals reflects the transition toward broader predictive and computational applications. Recent articles in high-impact journals deserve attention even when their citation counts remain low. Marsilio et al. published spatially explicit multicellular digital twins in Cancer Research in 2025. The models were calibrated with in vivo prostate and renal cancer data and reproduced angiogenesis, bone resorption, and treatment responses. The record had accumulated only one citation at the time of export (33)Morris et al. integrated automated bone imaging, more than 1,000 CT radiomics features, clinical variables, and serum biomarkers in Clinical Cancer Research. Model derivation and validation were conducted across two phase III trials. Imaging improved risk stratification but did not improve patient-specific survival-time prediction (42).These findings should not be interpreted as evidence of limited scientific value. They illustrate citation latency in papers published during 2025–2026. During such a short citation window, multicenter design, independent testing, reader benefit, real-world evaluation, and trial-level validation are more informative than citation counts. Journal prestige, however, is not a substitute for topical relevance. A paper in a highly ranked journal may address bone metastasis without making AI its principal method. Conversely, technically important work may appear in a specialist or non-core journal (43).

The author analysis showed that productivity was concentrated within a small number of collaborative groups. Publication output nevertheless did not always indicate durable scientific influence. Cao, Lin, Zeng, and Man formed one of the most productive recent clusters, with approximately 18 publications per author after name harmonization. These investigators constituted a closely connected network rather than four independent research programs. The technical progression within this group was relatively coherent. Lin et al., with Cao and Man as co-authors, compared VGG, ResNet, DenseNet, and a self-developed classifier for thoracic SPECT analysis. An area under the receiver operating characteristic curve of 0.9933 was reported using an augmented test dataset (44). Li et al. subsequently developed a convolutional neural network for classifying bone scans in patients with lung cancer. Lin, Zeng, Man, and Cao were included among the authors, and average classification accuracy was 0.7392 (45). Sustained collaboration allowed the group to refine model architectures and extend its work from classification towards segmentation. Reported performance must still be interpreted in relation to study design. High accuracy in augmented data or an internal test cohort does not guarantee similar performance in independent clinical populations. Multicenter external validation remains essential for establishing robustness and clinical applicability. Bibliometric productivity should not be equated with research quality, scientific leadership, or clinical significance. Publication counts primarily describe scholarly output and do not distinguish the methodological rigor or clinical relevance of individual studies. They also do not fully account for differences in first, corresponding, senior, and supporting authorship. Edenbrandt’s contribution followed a different pattern. Scientific influence was built through sustained methodological development rather than publication volume alone. Twelve publications were attributed to this author, with 353 cumulative citations and an H-index of 8. This research program advanced from ANN-based interpretation of planar bone scans in 2006 to CAD, multicenter calibration, and quantitative assessment of skeletal tumor burden (39, 41). Later studies established BSI as an imaging biomarker for treatment-response evaluation in metastatic prostate cancer (46). The work was subsequently extended to automated whole-skeleton analysis using deep learning (27). This continuity helps explain why a modest publication portfolio can exert substantial long-term influence.

Citation-burst and local-citation analyses further clarified the intellectual development of the field. Early investigations sought to reduce observer variability, standardize tumor-burden assessment, and establish clinically meaningful imaging endpoints. Dennis et al. validated BSI as a quantitative biomarker of skeletal metastatic burden and treatment response (47). Horikoshi et al. and Nakajima et al. then highlighted the importance of calibration and multicenter data for maintaining model reliability across populations (39, 41). These studies established persistent problems in medical AI. They include reference-standard definition, representation of metastatic burden, and preservation of performance after external deployment. The adoption of deep learning shifted the field from manually specified imaging parameters towards data-driven representation learning. CNNs and segmentation architectures offered new approaches to lesion detection and quantitative characterization (48). In bone-metastasis research, these methods were applied to spinal MRI, bone scintigraphy, CT, and radiomics. The resulting models supported automated classification, segmentation, and phenotype characterization (28–30, 49) Compared with early CAD systems, these approaches can extract richer spatial information from complex images. Their clinical value nevertheless remains conditional on external validation across heterogeneous patients, scanners, acquisition protocols, and care settings.

### 4.3 Research hotspots and thematic evolution

The thematic evolution observed in this study is best interpreted as a change in clinical questions rather than a sequence of fashionable algorithms. AI applications have gradually moved beyond lesion detection toward broader disease characterization and clinical decision support. This transition represents a response to increasingly complex clinical requirements in metastatic disease management. Early AI applications should be interpreted as supportive decision-making tools designed to enhance imaging consistency rather than as replacements for specialist interpretation (50). A subsequent thematic transition was observed from lesion identification toward quantitative evaluation of skeletal tumor burden. The development of imaging-derived biomarkers represented an important conceptual change because metastatic involvement was no longer considered only as a binary diagnostic outcome. Disease burden could be measured longitudinally and potentially linked with treatment response and clinical outcomes. The BSI became one of the representative quantitative approaches by converting whole-body skeletal imaging into a reproducible biomarker (47, 51). Similar concepts were later explored in breast cancer bone metastasis, where automated skeletal burden assessment showed potential associations with skeletal-related events and clinical outcomes, although further validation was required before clinical thresholds could be established (52).The increasing availability of large-scale imaging datasets subsequently shifted research attention toward radiomics, deep learning, and predictive modeling. In the present bibliometric analysis, these topics represented major emerging research clusters. It was suggested that AI applications have expanded from extracting visible imaging features toward learning complex representations associated with disease behavior. Radiomics approaches attempt to capture quantitative imaging phenotypes related to tumor heterogeneity, while deep learning models enable automated feature extraction without relying entirely on predefined imaging characteristics (53). The clinical significance of radiomic and deep-learning features depends on methodological robustness, external validation, and biological interpretation rather than model performance alone.

More recent thematic changes identified in this study indicate a transition toward AI-supported clinical decision-making through multimodal information integration. More recent studies in the dataset also included prognostic modeling, electronic health record analysis, natural language processing, and early applications of large language models. However, these language-based applications represented only a small component of the literature compared with imaging, machine learning, deep learning, and radiomics (54, 55). This development reflects the reality that management of bone metastasis requires more than imaging diagnosis. Treatment selection and clinical planning depend on survival estimation, fracture risk, neurological complications, systemic treatment history, and individual patient characteristics. Machine-learning-based prognostic models have therefore been explored to support individualized decision-making. Thio et al. developed machine-learning models for predicting short-term and one-year survival after surgery for extremity metastases, demonstrating the potential of AI-based risk estimation in surgical oncology settings (55). Predictive performance should be interpreted together with calibration, clinical utility, and external applicability rather than discrimination metrics alone.

Language-based AI represented a small but emerging component of the literature. The available studies primarily focused on information extraction, clinical data structuring, cohort construction, and knowledge accessibility rather than autonomous clinical decision-making. Kanemaru et al. developed a fine-tuned LLM for identifying progressive bone metastasis from unstructured radiology reports and achieved high classification accuracy with substantially reduced processing time compared with manual annotation, highlighting its potential for automated clinical data organization and cohort construction (11). Beyond information extraction, Li et al. evaluated several advanced LLMs for bone metastasis and cancer-induced bone pain-related questions from research, clinical, and patient perspectives, showing that these systems could provide accessible medical information while also revealing limitations related to bias, interpretability, and accountability (55). These findings indicate that AI applications in bone metastasis are gradually extending beyond imaging interpretation toward clinical information integration and patient-oriented support. Large language models (LLMs) are now being used to structure reports, identify metastatic sites, assemble cohorts, and support referral review. Tang et al. compared DeepSeek-R1, DeepSeek-V3, GPT-o3, and Gemini 2.5 Pro in a Chinese multicenter workflow. Clinical-logic-guided prompting converted bone-scintigraphy narratives into structured data without task-specific fine-tuning. The human-in-the-loop workflow reduced manual processing time by 74.5–82.6% and supported construction of an atlas involving 18,331 patients (31). Corso et al. used Llama 3.1 8B to retrieve variables from electronic health records. Accuracy for bone-metastasis status was 0.85 (32). These studies demonstrate the feasibility of language models for clinical data organization, but they should not be interpreted as evidence that LLMs or autonomous AI agents have become established research paradigms in bone metastasis. Autonomous systems capable of multistep clinical reasoning were rare in the present corpus. Marsilio et al. instead developed spatially explicit multicellular agent-based models as in silico digital twins of bone metastasis. These models simulated angiogenesis, bone resorption, and responses to cabozantinib and zoledronic acid (33). This work marks an important move towards mechanistic simulation. It should also be emphasized that mechanistic agent-based models are conceptually distinct from LLM-based autonomous or semi-autonomous clinical AI agents. Current LLM-based approaches should be considered complementary tools rather than autonomous decision-making systems, as reliable clinical deployment still requires domain-specific validation, appropriate human oversight, and integration with established oncological workflows. Collectively, AI research in bone metastasis is moving from answering “where is the lesion?” toward addressing more clinically relevant questions regarding disease progression, individualized risk assessment, and treatment optimization. However, the thematic evolution identified by the present bibliometric analysis should not be interpreted as a direct comparison of methodological quality or clinical effectiveness across different AI approaches. The included literature encompassed heterogeneous tasks, including detection, classification, segmentation, radiomics, prognostic modeling, multimodal prediction, and language-based applications, with substantial differences in dataset characteristics, validation strategies, outcome definitions, and reported performance metrics. A formal cross-study comparison of methodological rigor, model performance, and clinical utility would therefore require predefined study-level extraction criteria and task-specific quality-assessment frameworks within a dedicated systematic review. The present analysis instead focused on the structural and thematic evolution of the field, while methodological quality and clinical significance were interpreted cautiously.

### 4.4 Clinical translation, unresolved challenges, and future directions

Despite substantial progress in artificial intelligence applications for bone metastasis, the transition from research development to routine clinical implementation remains limited. Most current AI systems are designed for specific tasks, such as lesion detection, segmentation, or outcome prediction, and are commonly evaluated using retrospective datasets. Although high diagnostic or predictive performance can demonstrate technical feasibility, clinical adoption requires evidence of robustness, reproducibility, and measurable improvement in patient care. Interpretability and reproducibility should be considered as related but distinct requirements. Explainability methods may help clinicians identify which image regions or clinical variables contribute to model outputs, but post hoc explanations do not necessarily demonstrate that a model has learned clinically valid or generalizable relationships (56).Reproducibility additionally depends on transparent reporting of cohort selection, preprocessing, feature extraction, model architecture, hyperparameter tuning, validation procedures, and software environments (57).Previous evaluations of medical AI studies have highlighted that many models remain restricted by limited external validation, insufficient reporting transparency, and uncertainty regarding real-world clinical impact (58, 59). Performance obtained from internal or single-center retrospective cohorts may not be maintained when models are transferred to independent institutions or prospective clinical settings. Future studies should therefore progress from internal validation to geographically and temporally independent external validation, followed by prospective multicenter evaluation under real-world clinical conditions. Such evaluation should assess not only discrimination or segmentation performance, but also calibration, clinical utility, workflow impact, and clinically meaningful outcomes (35).

One of the major challenges for future implementation is the establishment of reliable and representative data infrastructures. Bone metastasis management involves heterogeneous information derived from multiple sources, including medical imaging, pathological assessment, molecular profiling, electronic health records, and longitudinal treatment information. However, these datasets are often fragmented across institutions, acquired using different protocols, and affected by variations in patient populations and clinical workflows. Such heterogeneity may reduce model generalizability when algorithms are transferred from development cohorts to external clinical environments. Generalizability may be further affected by differences in primary tumor type, disease burden, demographic composition, treatment exposure, referral patterns, and healthcare settings. Imaging-based models face additional variation related to scanner manufacturers, acquisition protocols, reconstruction parameters, image resolution, radiotracers, and post-processing procedures across CT, MRI, PET/CT, and bone scintigraphy. Models developed within relatively homogeneous datasets may therefore capture institution- or device-specific characteristics that do not generalize to other clinical environments. Dataset heterogeneity is also closely related to potential algorithmic bias (60).Retrospective clinical datasets may underrepresent particular patient groups, cancer types, disease stages, or healthcare settings. Selection bias may arise when datasets are derived from patients undergoing specific imaging examinations, specialist referral, surgery, or other predefined clinical pathways. Measurement bias may additionally be introduced by differences in reference standards, including radiology reports, manual annotations, pathological confirmation, follow-up imaging, or clinical consensus. Future studies should therefore report cohort composition, annotation procedures, missing data, and performance across clinically relevant subgroups to identify potential sources of systematic model error (60, 61).

The development of multicenter datasets, standardized data frameworks, and robust validation strategies will therefore be essential for improving the reliability of AI systems in oncology. The importance of diverse and representative datasets has been emphasized as a fundamental requirement for safe clinical AI deployment (62, 63) Importantly, multicenter evaluation should not be regarded solely as a means of increasing sample size. It should also test model stability across different patient populations, imaging systems, acquisition protocols, and clinical environments, with subgroup-specific performance reported where clinically relevant. Beyond data limitations, the clinical value of AI requires evaluation beyond conventional performance metrics. Many current studies primarily report accuracy, area under the curve, or segmentation scores, while relatively few assess whether AI systems improve clinical decision-making or patient outcomes. In oncology practice, the usefulness of an AI model depends on whether it can assist clinicians in complex scenarios, including risk assessment, treatment selection, response evaluation, and follow-up planning (64, 65). Prospective clinical evaluation and workflow-based assessment will therefore be required to determine whether AI provides meaningful advantages compared with existing clinical approaches (66, 67). Clinical implementation should also be considered a continuous process rather than the endpoint of model development. Changes in patient characteristics, imaging protocols, treatment strategies, or clinical documentation may lead to performance drift after deployment (68). Prospective implementation studies should therefore incorporate performance monitoring, predefined procedures for identifying model failure, and recalibration or updating when clinically appropriate.

The following perspective should be distinguished from the bibliometric findings of the present study. Disease-specific AI agents did not emerge as a dominant research theme in our corpus, and evidence for LLM-based agentic systems in bone metastasis remains limited. Recent language-based applications identified in the dataset, together with broader developments in medical AI, provide a rationale for considering how more integrated systems might be evaluated in future research (31, 55). One possible research direction is that future development of AI in bone metastasis may gradually move beyond isolated task-specific models toward more integrated disease-specific clinical AI agents. Current systems are predominantly designed for individual tasks, such as lesion detection, segmentation, prognostic prediction, or information extraction. However, the clinical management of bone metastasis requires continuous integration of heterogeneous information and sequential decision-making across diagnosis, risk assessment, treatment, and follow-up. These possibilities remain conceptual in the context of bone metastasis and require task-specific validation before clinical value can be inferred.

From a prospective perspective, AI agents may provide a potential computational architecture for this purpose by combining large language models, retrieval-augmented generation, external knowledge resources, and specialized computational tools to support multistep reasoning and evidence-based decision assistance. Such architectures could theoretically support the integration of imaging, pathology, molecular profiles, electronic health records, treatment history, and longitudinal follow-up data (69–74). Potential applications may include identifying patients at high risk of developing bone metastasis, evaluating metastatic progression, estimating skeletal-related event and fracture risk, monitoring treatment response, predicting prognosis, and supporting multidisciplinary treatment planning. Emerging language-based applications in bone metastasis already suggest that large language models can contribute to clinical information extraction and knowledge support (55). Evidence supporting RAG specifically in bone metastasis remains scarce. Drawing on the broader medical AI literature, RAG could be investigated as a mechanism for grounding model outputs in curated biomedical evidence. Whether such approaches improve factual reliability or clinical utility in bone metastasis requires direct evaluation (75). A clear distinction is required between clinical decision-support tools and autonomous or semi-autonomous AI agents. Most AI applications identified in the present corpus remain task-specific decision-support systems. They perform predefined functions such as lesion detection, segmentation, prognostic prediction, information extraction, or evidence organization, while interpretation and subsequent clinical actions remain under clinician control. In contrast, agentic AI systems may involve multistep task planning, retrieval of external information, tool selection and use, integration of intermediate outputs, and potentially initiation of downstream actions with a greater degree of operational autonomy. These systems should be developed around clearly defined clinical scenarios rather than technology-driven objectives and should function as supportive clinical infrastructures rather than autonomous decision-makers (76, 77). These systems should be developed around clearly defined clinical scenarios rather than technology-driven objectives and should function as supportive clinical infrastructures rather than autonomous decision-makers.

The successful development of bone metastasis-specific AI agents will depend on reliable data infrastructure, systematic evaluation, and continuous clinical oversight. Clinicians should retain authority over clinically consequential decisions, particularly those involving diagnosis, fracture-risk assessment, prognosis, treatment planning, or referral. AI outputs should be reviewable, and clinical workflows should allow human verification, correction, or override when information is incomplete, uncertain, or inconsistent with the broader clinical context. Responsibility for final clinical decisions should remain clearly defined within the healthcare workflow (78). Public databases, multicenter clinical cohorts, imaging repositories, digital pathology resources, molecular profiling platforms, and longitudinal clinical data will need to be integrated within standardized and interoperable frameworks. Different language models and agent architectures should be evaluated not only for predictive accuracy, but also for domain-specific reasoning, evidence retrieval, tool-use reliability, interpretability, robustness, and clinical utility. For agentic systems, evaluation should additionally consider evidence-retrieval accuracy, hallucination and omission, incorrect tool selection or execution, uncertainty handling, consistency across repeated tasks, and the potential clinical consequences of erroneous downstream actions. Increasing system autonomy should therefore be accompanied by increasingly rigorous requirements for validation, monitoring, transparency, and clinical accountability (79). AI deployment should therefore be regarded as an ongoing process rather than a one-time model development task, with continuous monitoring, updating, and adaptation as clinical practice and available evidence evolve (80, 81) Rather than replacing oncologists or radiologists, future disease-specific AI agents may serve as collaborative systems that organize complex information, provide evidence-grounded reasoning support, and facilitate personalized management. Their ultimate value will depend on rigorous clinical validation and demonstrated benefit within real-world oncology workflows. Accordingly, the near-term clinical role of AI in bone metastasis is more appropriately considered clinician-supervised decision support, while autonomous clinical decision-making remains an investigational direction requiring substantially stronger evidence and governance.

### 4.5 Limitations

This study has several limitations. First, publications were retrieved from WoSCC, PubMed, and Scopus. Although the use of three databases increased coverage, relevant studies indexed in other sources may have been missed. The restriction to English-language articles and reviews may also have reduced the representation of regional research. Second, bibliometric maps are influenced by analytical thresholds, term merging, clustering parameters, and the interpretation of cluster labels. Keyword normalization reduced artificial fragmentation but may also have combined terms with partially different meanings. Emerging or declining themes should therefore be interpreted cautiously. Their position may reflect limited sample size or recent publication rather than a true reduction in scientific relevance. Third, citation-based indicators are affected by time. Older publications have had more opportunity to accumulate citations, whereas recent high-quality studies may be underestimated. Citation counts may also reflect the general popularity of a method rather than its specific contribution to bone metastasis. Forth, the 2026 dataset covered only January 1 to June 26 and therefore does not represent a complete publication year. Although these records were included to capture recent developments, annual output for 2026 should not be directly compared with publication counts from complete calendar years. Finally, this analysis describes the structure and evolution of the literature but does not directly evaluate the methodological quality, risk of bias, or clinical performance of individual AI models. A highly cited or frequently published topic is not necessarily supported by strong clinical evidence.

## 5 Conclusion

In summary, artificial intelligence research in bone metastasis has expanded rapidly over the past two decades and has evolved from computer-assisted image interpretation and quantitative skeletal assessment toward deep learning, radiomics, prognostic modeling, and emerging language-based applications. Imaging remains the dominant research foundation, while recent studies increasingly emphasize patient-level prediction, multimodal information integration, and clinical data organization. Despite these advances, translation into routine clinical practice remains limited by data heterogeneity, insufficient external validation, limited prospective evidence, and uncertainty regarding real-world clinical benefit. Future research should therefore prioritize representative multicenter datasets, rigorous and transparent validation, prospective workflow-based evaluation, and clinically meaningful endpoints. More integrated disease-specific AI systems, potentially incorporating language models and evidence-retrieval components, may represent a longer-term research direction rather than an established trajectory of the current literature. Their clinical value will require rigorous validation, human oversight, and demonstrated improvement in real-world patient care.

## Data Availability

The original contributions presented in the study are included in the article/**Supplementary Material**. Further inquiries can be directed to the corresponding authors.
