# Supplementary material for: Mapping the evolution of artificial intelligence applications in bone metastasis: a bibliometric and visualization analysis
Source: Front Oncol. 2026 Oct 21;16:1971840. doi: 10.3389/fonc.2026.1971840 (PMC13730961; doi:10.3389/fonc.2026.1971840)
Supplement: Supplementary file 1 [file Table1.docx]

**Supplementary Material**

**Supplementary Table 1. Articles excluded for not meeting the inclusion criteria (n = 384)**

| **Reasons for exclusion** | **Quantity** | **Percentage** | **Explanation** |
| --- | --- | --- | --- |
| Bone metastasis was not confirmed from the title or keywords | 118 | 30.7% | The title, abstract keywords, or external screening information indicated that the record did not address bone metastasis together with an AI/ML method. Bone metastasis was not an actual analytical target. |
| Other diseases or metastatic sites were the primary research focus | 48 | 12.5% | The study mainly investigated another disease or another metastatic site; the title, abstract, and keywords did not support bone metastasis as the study target. |
| Bone metastasis and an eligible AI/ML method were not simultaneously supported | 192 | 50% | Available title, abstract, keyword, or external information did not establish both a bone-metastasis research target and direct application of an AI/ML method to that task. |
| Bone metastasis was not confirmed from the title or keywords | 22 | 5.7% | The title or keywords pointed to another disease or metastatic site, and bone metastasis could not be confirmed as the research target. |
| Publication restrictions were not met | 3 | 0.8% | The record did not satisfy the predefined year, English-language, or document-type requirement (Article or Review). |
| Bone metastasis was studied, but no explicit AI/ML method was identified | 1 | 0.3% | The record addressed bone or skeletal metastasis but did not report a clearly defined AI-related method directly applied to a bone-metastasis task. |

**Supplementary Table S2.** Country and region name standardization rules

| **Label** | **Replace By** |
| --- | --- |
| Peoples r China | China |
| Taiwan | China |
| Korea | South Korea |
| Republic of Korea | South Korea |
| Turkey | Turkiye |
| United States | USA |
| England | United Kingdom |

**Supplementary Table S3.** Institution name standardization rules

| **Label** | **Replace By** |
| --- | --- |
| air force med univ | Air Force Medical University |
| alma mater studiorum bologna univ | University of Bologna |
| alma mater studiorum univ bologna | University of Bologna |
| anton van leetiwenhoek hosp | Antoni van Leeuwenhoek Hospital |
| anton van leeuwenhoek hosp | Antoni van Leeuwenhoek Hospital |
| antoni van leeuwenhoek hosp | Antoni van Leeuwenhoek Hospital |
| antoni von leeuwenhoek hosp | Antoni van Leeuwenhoek Hospital |
| ap–hp.nord-université paris cité | AP-HP.Nord – Université Paris Cité |
| asia univ | Asia University |
| case western reserve univ | Case Western Reserve University |
| case western reserve univ cwru | Case Western Reserve University |
| catholic univ louvain | Catholic University of Louvain (UCLouvain) |
| charles sturt univ | Charles Sturt University |
| china med univ hosp | China Medical University Hospital |
| china medical university (shenyang) | China Medical University |
| china medical university (taiwan) | China Medical University Taiwan |
| chinese acad med sci & peking union med coll | Chinese Academy of Medical Sciences & Peking Union Medical College |
| ctr res & technol hellas | Centre for Research and Technology Hellas (CERTH) |
| ctr res & technol hellas certh | Centre for Research and Technology Hellas (CERTH) |
| exini diagnost ab | Exini Diagnostics AB |
| exini diagnostics – a lantheus company | Exini Diagnostics AB |
| exini diagnostics ab | Exini Diagnostics AB |
| fdn irccs ist nazl tumori | Fondazione IRCCS Istituto Nazionale dei Tumori, Milan |
| fdn irccs ist nazl tumori milano | Fondazione IRCCS Istituto Nazionale dei Tumori, Milan |
| fondazione irccs istituto nazionale dei tumori | Fondazione IRCCS Istituto Nazionale dei Tumori |
| fondazione irccs istituto nazionale dei tumori di milano | Fondazione IRCCS Istituto Nazionale dei Tumori |
| fondazione ircss istituto nazionale dei tumori di milano | Fondazione IRCCS Istituto Nazionale dei Tumori |
| fourth mil med univ | Air Force Medical University |
| fudan univ | Fudan University |
| fujan med univ | Fujian Medical University |
| fujian med univ | Fujian Medical University |
| gansu prov canc hosp | Gansu Provincial Cancer Hospital |
| gansu prov hosp | Gansu Provincial Hospital |
| gansu prov peoples hosp | Gansu Provincial Hospital |
| gansu prov tumor hosp | Gansu Provincial Cancer Hospital |
| gansu prov tumour hosp | Gansu Provincial Cancer Hospital |
| gen hosp northern theater command | General Hospital of Northern Theater Command |
| gen univ hosp | General University Hospital |
| gen univ hosp prague | General University Hospital |
| gothenburg univ | University of Gothenburg |
| grigore t popa univ med & pharm | Grigore T. Popa University of Medicine and Pharmacy |
| grigore t popa univ med & pharm iasi | Grigore T. Popa University of Medicine and Pharmacy |
| harvard med sch | Harvard Medical School |
| harvard univ | Harvard University |
| heidelberg univ hosp | Heidelberg University Hospital |
| hosp civils lyon | Hospices Civils de Lyon |
| hospices civils de lyon | Hospices Civils de Lyon |
| irccs ist romagnolo studio tumori dino amadori | IRST IRCCS 'Dino Amadori' |
| irccs ist romagnolo studio tumori irst dino amador | IRST IRCCS 'Dino Amadori' |
| ist nazl tumori irccs | Fondazione IRCCS Istituto Nazionale dei Tumori |
| ist sci romagnolo studio & cura tumori irst srl | IRST IRCCS 'Dino Amadori' |
| johns hopkins hospital | The Johns Hopkins Hospital |
| johns hopkins sch med | Johns Hopkins University School of Medicine |
| johns hopkins univ | Johns Hopkins University |
| johns hopkins univ hosp | The Johns Hopkins Hospital |
| johns hopkins university | Johns Hopkins University |
| johns hopkins university school of medicine | Johns Hopkins University School of Medicine |
| kanazawa univ | Kanazawa University |
| katholieke univ leuven | KU Leuven |
| ku leuven lib | KU Leuven |
| ku leuven univ leuven | KU Leuven |
| lanzhou univ | Lanzhou University |
| lund univ | Lund University |
| maastricht univ | Maastricht University |
| masatyk univ brno | Masaryk University |
| massachusetts gen hosp | Massachusetts General Hospital |
| nanchang univ | Nanchang University |
| natl taiwan univ | National Taiwan University |
| natl taiwan univ hosp | National Taiwan University Hospital |
| naval med univ | Naval Medical University |
| navy med univ | Naval Medical University |
| nord univ paris cite | AP-HP.Nord – Université Paris Cité |
| northeastern university (china) | Northeastern University (China) |
| northeastern university (united states) | Northeastern University (United States) |
| northwest minzu univ | Northwest Minzu University |
| osaka univ | The University of Osaka |
| peking union med coll & chinese acad med sci | Chinese Academy of Medical Sciences & Peking Union Medical College |
| peking univ | Peking University |
| peking univ first hosp | Peking University First Hospital |
| politecn milano univ | Politecnico di Milano |
| politecnico di milano | Politecnico di Milano |
| qingdao univ | Qingdao University |
| radiodynam healthcare | Radiodynamic Healthcare |
| radiodynamic healthcare | Radiodynamic Healthcare |
| sahlgrens univ hosp | Sahlgrenska University Hospital |
| sapienza univ rome | Sapienza University of Rome |
| seoul natl univ | Seoul National University |
| seoul natl univ hosp | Seoul National University Hospital |
| shandong first med univ | Shandong First Medical University |
| shandong first med univ & shandong acad med sci | Shandong First Medical University |
| shandong first med univ shandong acad med sci | Shandong First Medical University |
| shanghai jiao tong univ | Shanghai Jiao Tong University |
| sichuan univ | Sichuan University |
| spectrum dynam med | Spectrum Dynamics Medical |
| spectrum dynamics medical | Spectrum Dynamics Medical |
| st annes univ hosp | St. Anne's University Hospital Brno |
| st annes univ hosp brno | St. Anne's University Hospital Brno |
| sun yat sen univ | Sun Yat-sen University |
| taipei med univ | Taipei Medical University |
| third mil med univ | Army Medical University |
| tianjin med univ | Tianjin Medical University |
| univ bologna | University of Bologna |
| univ campus biomed roma | Campus Bio-Medico University of Rome |
| univ catholique louvain ucl | Catholic University of Louvain (UCLouvain) |
| univ catholique louvain uclouvain | Catholic University of Louvain (UCLouvain) |
| univ claude bernard lyon 1 | Claude Bernard University Lyon 1 |
| univ gen hosp | University Hospital of Heraklion |
| univ gothenburg | University of Gothenburg |
| univ hosp heidelberg | Heidelberg University Hospital |
| univ lorraine | University of Lorraine |
| univ lyon | University of Lyon |
| univ maastricht | Maastricht University |
| univ med ctr utrecht | University Medical Center Utrecht |
| univ milan | University of Milan |
| univ new south wales | UNSW Sydney |
| univ rome campus biomed | Campus Bio-Medico University of Rome |
| univ rome sapienza | Sapienza University of Rome |
| univ thessaly | University of Thessaly |
| univ toronto | University of Toronto |
| univ utrecht | Utrecht University |
| università degli studi di milano | University of Milan |
| université claude-bernard lyon i | Claude Bernard University Lyon 1 |
| université de lorraine | University of Lorraine |
| université de lyon | University of Lyon |
| unsw | UNSW Sydney |

**Supplementary Table S4.** Unified keyword standardization rules

| **Label** | **Replace By** |
| --- | --- |
| ai | artificial intelligence |
| artificial intelligence (ai) | artificial intelligence |
| artificial neural networks | artificial neural network |
| bone metastases | bone metastasis |
| bone metastasis (bm) | bone metastasis |
| skeletal metastasis | bone metastasis |
| skeletal metastases | bone metastasis |
| metastatic bone disease | bone metastasis |
| tumor bone metastasis | bone metastasis |
| bone marrow metastasis | bone metastasis |
| spinal metastases | spinal metastasis |
| spine metastases | spinal metastasis |
| spine metastasis | spinal metastasis |
| thoracic spinal metastases | spinal metastasis |
| bone scintigram | bone scintigraphy |
| bone scintigrams | bone scintigraphy |
| skeletal scintigraphy | bone scintigraphy |
| scintigraphy | bone scintigraphy |
| bsi | bone scan index |
| cad | computer-aided diagnosis |
| computer-aided diagnosis (cad) | computer-aided diagnosis |
| computer-assisted diagnosis | computer-aided diagnosis |
| computer-aided detection (cad) | computer-aided detection |
| cnn | convolutional neural network |
| convolutional neural networks | convolutional neural network |
| ct | computed tomography |
| computed tomography (ct) | computed tomography |
| mri | magnetic resonance imaging |
| machine learning (ml) | machine learning |
| machine-learning | machine learning |
| multiple myeloma (mm) | multiple myeloma |
| nsclc | non-small cell lung cancer |
| pet-ct | PET/CT |
| pet/ct | PET/CT |
| positron-emission tomography | positron emission tomography |
| prediction models | prediction model |
| predictive model | prediction model |
| prostate cancer (pca) | prostate cancer |
| radiation therapy | radiotherapy |
| segmentation | image segmentation |
| lesion segmentation | image segmentation |
| medical image segmentation | image segmentation |
| single-photon emission computed tomography | single photon emission computed tomography |
| spect | single photon emission computed tomography |
| whole-body mri | whole-body magnetic resonance imaging |
